# Over-detection and over-surveillance in breast screening: current status and the potential for artificial intelligence optimisation

**DOI:** 10.1186/s13244-025-02160-w

**Published:** 2025-12-12

**Authors:** Siyu Wang, Jingyan Liu, Linlin Song, Wen Wen, Juan Huang, Yulan Peng

**Affiliations:** 1 Department of Medical Ultrasound, West China Hospital, Sichuan University, Chengdu, China; 2 Department of Radiology, West China Hospital, Sichuan University, Chengdu, China

**Keywords:** Artificial intelligence, Breast cancer screening, Over-detection, BI-RADS, Risk stratification

## Abstract

**Abstract:**

Breast screening reduces cancer-specific mortality but can also precipitate avoidable harms through over-detection of benign abnormalities and subsequent over-surveillance. Across mammography and digital breast tomosynthesis (DBT), ultrasound and magnetic resonance imaging (MRI), gains in sensitivity are often offset by reduced specificity, driving false-positive recalls, benign-biopsy burden and resource strain. Within breast imaging reporting and data system (BI-RADS)–guided decision-making, Category 3 and Category 4A trigger short-interval follow-up or biopsy despite low event rates, amplifying anxiety and cost. Artificial intelligence (AI) offers a practical route to mitigate these drawbacks. Prospective and real-world studies indicate that AI-assisted reading can maintain or improve cancer detection while lowering recall rates and workload. AI models also support finer risk stratification—particularly for BI-RADS 4 lesions—thereby reducing unnecessary interventions. This review synthesises evidence on the performance and limitations of mainstream screening technologies, delineates the multidimensional impact of over-detection, and evaluates the capacity of AI to rebalance sensitivity and specificity, optimise follow-up intervals and support risk-adapted workflows. A patient-centred, evidence-driven strategy that integrates validated AI with clearly defined decision thresholds and effective patient-provider communication can maximise benefit while minimising harm.

**Critical relevance statement:**

This review critically evaluates the causes and consequences of over-detection and over-surveillance in breast cancer screening and highlights how AI can advance radiologic decision-making through improved lesion stratification and more efficient, personalised follow-up strategies.

**Key Points:**

- BI-RADS thresholds largely drive over-detection; refining downgrade rules for 3 and tightening biopsy in 4A may reduce unnecessary interventions without compromising cancer detection.
- Over-detection imposes burdens: unnecessary imaging and biopsies, psychosocial distress, economic costs, and environmental impact; its reduction enhances efficiency and patient safety.
- AI-assisted screening maintains or improves cancer detection while reducing recall rates and workload; it also enables risk-adapted management of BI-RADS 4A lesions, avoiding low-value procedures.

**Graphical Abstract:**

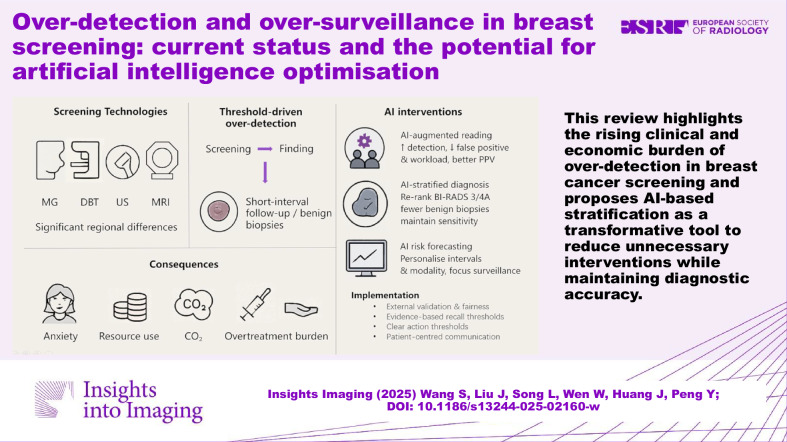

## Introduction

Breast cancer remains one of the most prevalent malignancies among women globally and a leading cause of cancer-related mortality [1]. Contemporary randomised and population-based evaluations indicate that organised mammography screening reduces breast cancer–specific mortality by approximately 20–30% [1–3]. However, as sensitivity increases across imaging modalities, specificity often declines, resulting in more false positives and cascading downstream consequences—unnecessary imaging, short-interval surveillance, and low-yield biopsies [4–7].

Over-detection and overdiagnosis are conceptually different, but frequently confused when discussing breast cancer screening. Overdiagnosis refers to the detection of indolent cancers that would never cause symptoms or death if left untreated [8], with literature estimates ranging from ~10% to more than 50%, depending on study design and methodology [9–12]. In contrast, this review focuses on over-detection, defined as positive imaging findings ultimately proven benign through pathology or long-term follow-up [13]. Here, false-positive imaging findings—abnormalities on screening that are not malignant—are considered the main clinical manifestation of over-detection. While overdiagnosis can lead to long-term concerns, the day-to-day burden in practice is driven more by false positives, unnecessary follow-up, and avoidable procedures [14, 15].

Crucially, the rate of over-detection is not dictated solely by the imaging modality itself but is largely governed by programmatic thresholds and management rules. Factors such as recall criteria, breast imaging reporting and data system (BI-RADS) management thresholds [16], and how sensitivity takes priority over specificity jointly determine the over-detection risk. Small shifts in recall criteria or BI-RADS cut-offs can translate into large-scale increases in follow-up procedures and benign biopsies at the population level. In particular, refining decision-making around BI-RADS 3 (≤ 2% likelihood of malignancy) and BI-RADS 4A (> 2%– ≤ 10%) is pivotal to containing over-detection while preserving cancer detection performance [16, 17].

Against this backdrop, artificial intelligence (AI), particularly deep-learning models, has emerged as a promising tool to mitigate these limitations [18, 19]. By enhancing lesion classification, improving risk estimation, and supporting workflow triage, AI offers the potential to reduce false positives, refine BI-RADS categorisation, and personalise follow-up strategies [20, 21]. Recent prospective trials and real-world deployments suggest that AI can maintain or even improve cancer detection rates while lowering recall rates and radiologist workload [22–24]. In light of these developments, it is crucial to comprehensively examine the current screening landscape and the burden of over-detection, as well as the emerging role of AI in addressing these challenges.

This review integrates the performance and limitations of mainstream screening techniques, with a focus on recall rates, BI-RADS decision thresholds, and the clinical consequences of over-detection. It also explores the emerging potential of AI in rebalancing sensitivity and specificity, simplifying follow-up pathways, and supporting more personalised, risk-adaptive screening strategies.

## Efficacy and limitations of global mainstream screening technologies

Global breast cancer screening strategies vary across regions, both in the choice of modalities and in downstream management; Table 1 compiles current average-risk guidelines issued by selected national task forces and medical societies in major countries/regions [25–37].

**Table 1:** Comparative summary of international breast cancer screening guidelines by key organisations

Country/region	Guideline name and year	Publishing organisation	Age and interval (average-risk)	Primary modality	Supplemental for dense breasts
Australia	*Early Detection of Breast Cancer—Position Statement* (2015; updated 2025-01-30) [25]	Cancer Australia	Invite 50–74 every 2 y; ages 40–49 and > 74 may attend by choice.	Mammography	Routine supplemental imaging is not endorsed for average-risk; DBT may increase detection, but the mortality benefit has not been established.
Canada	*Breast Cancer—Draft Recommendations* (2024) [26]	Canadian Task Force on Preventive Health Care (CTFPHC)	40*–*74: offer mammography every 2*–*3 y by preference; 50*–*74: suggest every 2*–*3 y; ≥ 75: suggest no screening.	Mammography	Supplemental MRI/US is not recommended for dense breasts at moderate risk (very low certainty).
China	*Screening and Early Diagnosis of Breast Cancer in China: A Practice Guideline* (2022) [27]	Chinese Anti-Cancer Association (CACA) / Chinese Breast Cancer Society	40*–*49 and 50*–*69: mammography every 2 y; 70*–*74: optional, if screened every 2*–*3 y	Mammography; DBT or DM acceptable	If the first mammogram shows dense breasts: add an ultrasound; MRI is not recommended as a routine supplement; ultrasound can be used when mammography is not feasible.
Europe	*European Guidelines on Breast Cancer Screening & Diagnosis* (current living guideline) [28]	ECIBC, European Commission	50—69 biennial (strong); 45*–*49 biennial/triennial (conditional); 70*–*74: avoid annual; triennial vs biennial debated	Mammography	For extremely dense breasts, EUSOBI recommends offering MRI with shared decision-making (varies by country) [29].
Germany	*S3-Leitlinie Mammakarzinom* (Version 5, 2025 consultation) [30]	Leitlinienprogramm Onkologie (DKG/DKH/AWMF)	National programme invites 50–69 biennial; early detection content integrated in the S3 guideline.	Mammography (programme; DBT discussed)	Dense-breast handling is detailed in S3; the programme remains mammography-based.
India	*Best Practice Guidelines for Breast Imaging – Part 1: Screening* (2022) [31]	Breast Imaging Society of India (BISI)/Ann. Natl. Acad. Med. Sci. (India)	≥ 40 annual screening recommended where mammography is available; resource-tailored guidance.	Mammography (preferred modality)	Ultrasound is mainly an adjunct for younger/dense breasts; ultrasound-only screening is generally not advised where mammography is available.
Japan	*JBCS Clinical Practice Guidelines—Screening & Diagnosis* (2022) [32]	Japanese Breast Cancer Society (JBCS)	≥ 40 biennial mammography in municipal/organised programmes	Mammography (organised screening)	Handheld ultrasound as an adjunct can be considered with quality control; ultrasound alone is not advised for population screening.
South Korea	*Korean Guideline for Breast Cancer Screening* (2015) [33]	Korean Medical Association/National Cancer Centre taskforce	40–69 biennial mammography; ≥ 70 selective by risk/preferences.	Mammography	No universal rule; in practice, supplemental ultrasound is commonly used for dense breasts
United Kingdom	*UK NSC breast screening policy* (2024) [34]	UK National Screening Committee	50*–*71 triennial; ≥ 71 self-referral	Mammography	No routine supplemental imaging solely for density in the national programme; UK NSC currently reviewing evidence on additional imaging for dense breasts [35].
United States	*Breast Cancer Screening—Final Recommendation* (2024) [36]	U.S. Preventive Services Task Force (USPSTF)	40–74 biennial screening.	Mammography (digital or DBT)	The evidence is insufficient to recommend for/against supplemental ultrasound or MRI after a negative mammogram.
United States	*Breast Cancer Screening & Diagnosis* (Version 2.2025) [37]	National Comprehensive Cancer Network (NCCN)	Begin at 40; annual screening generally supported (professional guideline; patient guideline aligns via risk-/preference-based cadence).	Mammography; DBT preferred when available	Consider supplemental imaging for heterogeneously or extremely dense breasts after risk assessment and shared decision-making.

Countries are listed in alphabetical order for clarity and neutrality. All entries are based on national clinical practice guidelines or official position statements, not programme operations documents

Because this review focuses on population (average-risk) screening, high-/very-high-risk pathways are not detailed in the table. In brief, major guidelines generally recommend annual MRI (±mammography) for women with ≥ 20–25% lifetime risk or specific syndromes (e.g. BRCA1/2, prior chest irradiation), with implementation details varying by country

### Mammography (breast X-ray) and digital breast tomosynthesis (DBT)

Mammography underpins most organised screening programmes, with well-established mortality reduction [2, 3]. However, in real-world United States practice, per-round recall rates approach 10–12%, yet only ~0.5% of those recalled are ultimately diagnosed with cancer—reflecting a low positive predictive value (PPV) [38]. Over a 10-year screening period, nearly half of all screened women will experience at least one false-positive result [39].

DBT, by reducing tissue overlap, has been associated with modest reductions in false positives. Large studies report recall rate improvements of up to 2.2% compared with digital mammography (DM) [40]. Data from the Breast Cancer Surveillance Consortium (BCSC) show that, over 10 years, the probability of ≥ 1 false-positive recall is slightly lower with DBT vs DM (annual screening: 50% vs 56%; biennial: 35.7% vs 38.1%), though cumulative short-interval follow-up and biopsy rates remain similar [41]. However, the benefit depends on local screening practice [42, 43].

In contrast, organised European programmes generally target lower recalls (~3–7%) [44–46]. National data from Ireland show that first-round recall rates rose from ~5.5% to 10% between 2000 and 2019, while recall rates in subsequent screening rounds increased from ~2.3% to 3% [44]. In low-recall Nordic programmes (baseline ~2–4%), data from the Malmö trial showed that DBT initially increased recalls (2.6% vs 0.8% with DM) but stabilised at ~1.5% in later years—highlighting that recall impact depends more on programme context and reader adaptation than the technology alone [47].

Taken together, small changes in what constitutes a recall or biopsy recommendation can move large numbers of women into short-interval follow-up or invasive assessment, with only marginal gains in PPV [48, 49]. This underscores the need for clearer diagnostic criteria and more risk-adapted management strategies to avoid over-detection.

### Ultrasound

In Western screening programmes, ultrasound typically supplements mammography for dense breasts or higher-risk women. In contrast, several Asian guidelines incorporate ultrasound more prominently into routine screening practice.

In average-risk women, the Japan strategic anti-cancer randomised trial showed that supplementing mammography with ultrasound increased cancer detection by approximately 54% (from 3 to 5 cancers per 1000 screens) but also produced 37 additional false-positive recalls and 27 additional biopsies per 1000 women screened [50]. Similarly, in the American College of Radiology Imaging Network (ACRIN) 6666 trial, supplementing mammography with ultrasound detected +3.7 per 1000 additional cancers (mostly node-negative early invasive) but increased biopsy rates by 5% [51]. In high-risk programmes such as the Cancer Screening Program in Urban China (CanSPUC) (across 72,250 high-risk women), 13.51% (9765) of first-round ultrasound examinations were rated BI-RADS 3–5 (mostly 3), with only 68 exams rated as BI-RADS 5. In other words, over 12% of the screened women had findings that were suspicious on ultrasound (BI-RADS 3 or 4) but turned out to be non-malignant on initial workup [52].

These findings highlight a key challenge in ultrasound-based screening: its high sensitivity for benign nodules (e.g. fibroadenomas, cysts) [53] often results in BI-RADS 3 or 4A assessment. When clinical pathways favour immediate biopsy over surveillance, benign lesions dominate pathology outcomes. While downgrading strategies (e.g. reclassifying selected BI-RADS 3 findings) have shown potential to reduce biopsy rates without compromising cancer detection [54], their long-term impact on interval cancer rates remains uncertain.

### Magnetic resonance imaging (MRI)

In population screening, MRI is not a universal test; it is reserved for women at substantially elevated risk and, in some programmes, for those with extremely dense breasts as an adjunct to mammography.

In women with extremely dense breasts, adding supplemental MRI to biennial mammography reduced the interval cancer rate from ~5.0 to ~2.5 per 1000 screens by detecting cancers that would otherwise present as interval cancers. However, programme evaluations report an absolute false-positive rate of ~80 per 1000 screens [55]. Because MRI is resource-intensive, variably available, and requires expert interpretation, its population-level benefit depends on risk-adapted selection and explicit management thresholds [4]. When selection and follow-up criteria are clear, additional detections can be achieved while limiting benign work-ups; in contrast, broad use with low thresholds expands callbacks and yields many low-value biopsies.

## BI-RADS-guided management and non-AI risk stratification

### BI-RADS-guided management

Beyond modality performance, whether a woman is recalled or biopsied is chiefly determined by interpretation thresholds codified in BI-RADS, which maps each category to an estimated probability of malignancy and a management recommendation (observation vs biopsy) [16].

BI-RADS 3 (“probably benign”). Defined by an expected malignancy ≤ 2%, this category is generally managed with short-interval surveillance rather than immediate biopsy. Large follow-up datasets show malignancy detection ~0.2% at 6 months and 0.39% at 2 years, rates comparable to BI-RADS 1–2 and similar to the background annual incidence of breast cancer (0.3–0.5%) [56]. Accordingly, several screening programmes now permit extending the initial follow-up interval to 12 months for concordant probably benign findings, while still recommending earlier review if any high-risk feature emerges [56, 57].

BI-RADS 4A (“low suspicion”). Defined as > 2%–≤ 10% likelihood of malignancy, this subcategory accounts for a large proportion of biopsy recommendations in breast imaging. In a single-centre retrospective analysis of 2433 biopsied cases, 44.5% (1083/2433) were classified as 4A, with a malignancy rate of ~8.8%, suggesting that most lesions were ultimately benign [58]. Broader literature further corroborates this: one multicentre cohort found that 68.4% of BI-RADS 4 lesions were 4A, and 70.9% of 4A lesions were confirmed benign [59], while another study reported BI-RADS 4A PPVs ranging from only 4–9% [60]. Unlike BI-RADS 4B, 4C, or 5, which justify biopsy owing to higher cancer odds, 4A is a pivotal category for minimising over-intervention.

Taken together, BI-RADS 3 and 4A are the main thresholds for over-monitoring and benign biopsy. Clarifying downgrade criteria for 3, standardising triggers for early reassessment, and tightening biopsy decision criteria in 4A, for example, by incorporating lesion-level features and clinical context, can curb over-detection without compromising cancer detection and can align daily practice with intended probability bands.

### Risk stratification (without AI)

Non‑AI risk stratification aims to concentrate supplemental imaging and closer follow‑up on women at elevated risk, thereby avoiding unnecessary interventions in low‑risk groups. In the BCSC framework, women with dense breasts and high-risk factors (e.g. family history, prior high-risk lesions) undergoing supplemental ultrasound had a cancer detection rate of ~5.5 per 1000 exams and a biopsy PPV of 15.0%, compared to ~1.3 per 1000 and 4.9% in lower-risk women [61].

Recent studies support broader adoption of risk-based screening. A 2025 randomised study found that adding polygenic risk scores and breast density improved stratification over demographic-only models [62]. However, practical barriers remain. Risk models require population-specific calibration. “High-risk” thresholds are inconsistently applied. Clinical integration is often lacking. For example, women with extremely dense breasts and a ≥ 1.67% 5-year risk show higher interval cancer rates under routine screening alone [63]. Aligning risk and density profiles with structured screening pathways could improve early detection and reduce false positives.

Imaging modality should be optimised after risk stratification. The UK BRAID trial (~9000 participants) found that contrast-enhanced mammography detected 15.7 cancers/1000 in women with dense breasts. This outperformed ultrasound (4.2) and matched MRI (15.0), while being more cost-effective [64]. In Canada, the PERSPECTIVE Integration & Implementation project (PERSPECTIVE I&I) tested large-scale risk assessment. It confirmed feasibility but revealed barriers such as questionnaire validation and density retrieval [65]. These gaps highlight the need for integrated, scalable, and personalised strategies. AI may help meet that need.

## Consequences of over-detection and over-surveillance

### Psychological impacts

False-positive mammogram results can cause lasting anxiety and decreases in quality of life [66, 67], as shown in longitudinal studies. In a BCSC analysis of over 3.5 million screenings, return rates fell from 77% after true-negative results to 61% after a false-positive requiring short-interval follow-up, and to 67% when biopsy was recommended, dropping further to 56% after two consecutive false positives [68]. Moreover, population data reveal a modest 3% decline in rescreening (relative risk (RR), RR ~ 0.97) following a false-positive, but those who did not return had more than threefold higher odds of interval cancer (adjusted odds ratio (OR) 3.19, 95% confidence interval (CI) 2.34–4.35) [69].

Anxiety in screening isn’t limited to the stress of false positives. Even in European programmes with low recall rates (~2–4%), ambiguity or low specificity can introduce another form of psychological burden—concern that cancers may be missed. While direct studies on ‘underdetection anxiety’ are lacking, broader evidence shows that screening errors and overscreening can undermine patient trust and elevate health-related anxiety [70, 71].

### Economic and resource burden

Over-detection drives substantial downstream spending through cascades of imaging, procedures, and follow-ups. In the U.S., these downstream screening costs reach about US$4 billion annually—US$2.8 billion from false-positive mammograms and US$1.2 billion from overdiagnosis [72]. These activities also strain limited resources like scanner availability and radiologist time, potentially delaying care for symptomatic patients.

Although not standard for average-risk screening, MRI use is expanding in women with dense breasts or elevated risk. In a large cohort study of insured women, each 1000 MRI screens (vs mammography) triggered roughly 50 extra imaging tests, 173 procedures, 130 outpatient visits, and 3 hospitalisations, adding about US$1404 in downstream costs per woman (95% CI, US$1172–1636) [73]. These data reinforce the need to restrict MRI to clearly defined high-risk populations.

### Environmental costs

Imaging carries a measurable carbon footprint that scales with avoidable recalls. Life-cycle assessments estimate per-scan emissions around ~17 kg carbon dioxide equivalent (CO₂e) for MRI, ~9 kg CO₂e for CT, and < 1 kg CO₂e for standard radiography/ultrasound [74]. System-level estimates suggest excess imaging accounts for ~4–30% of examinations and is associated with ~129 kT CO₂e/year in the United States Medicare population [75]. Reducing false positives, therefore, aligns clinical stewardship with environmental responsibility.

### Overtreatment and clinical sequelae

Over-detection in breast cancer screening often leads to overtreatment, particularly in the form of unnecessary biopsies and surgeries for benign lesions. After 10 years of annual screening, 7–12% of women undergo a biopsy that proves non-malignant [76]. While percutaneous biopsies are generally safe, open surgical biopsies carry complication rates of 4–6% for infection and 2–10% for haematoma [77].

These diagnostic pathways can escalate into surgical overtreatment. In large biopsy cohorts, BI-RADS 4A lesions account for up to 44.5% of biopsied nodules, yet the malignancy rate may be as low as 2.7% in women under 35 [58]. Such low-yield interventions can still expose patients to measurable harm, as even minimally invasive procedures may cause pain, bleeding, infection, or cosmetic sequelae. Reducing unnecessary biopsies and excisions through improved risk stratification, stricter biopsy thresholds, and standardised follow-up protocols is therefore essential to minimise avoidable harm and optimise patient safety.

## AI to reduce over-detection and optimise follow-up

AI, particularly deep learning, has shown promise in breast cancer screening by mitigating the subjectivity and variability of traditional risk stratification. Acting as an adjunct to radiologists, AI enhances lesion detection, classification, and individual risk estimation.

### AI-augmented screening: improving accuracy and efficiency

AI integration has consistently improved breast cancer screening accuracy while reducing false positives and workload. In Denmark’s national programme, AI-assisted double reading increased detection from 7.0 to 8.2 per 1000 screens, reduced false positives (2.39% → 1.63%) and recalls (~20.5%), improved PPV (22.6% → 33.6%), and raised the detection of small tumours (≤ 1 cm) from 36.6% to 44.9%, with a 33.5% reduction in reader workload [78]. Sweden’s mammography screening with artificial intelligence trial (MASAI trial) safely assigned ~90% of exams to a single reader with AI, achieving non-inferior cancer detection (6.1 vs 5.1 per 1,000 screens), identical false-positive rates (1.5%), improved recall PPV (24.8% → 28.3%), and a 44.3% workload reduction [79]. The population-based real-world AI mammography study (PRAIM) study (*n* = 463,094) in Germany showed increased detection (6.7 vs 5.7 per 1000 screens), stable recall (37.4 vs 38.3 per 1000), and improved PPV for both recalls (14.9% → 17.9%) and biopsies (59.2% → 64.5%) [22]. A multinational study by Google Health showed AI reduced false positives (US: −5.7%, UK: −1.2%) and false negatives (US: −9.4%, UK: −2.7%) while surpassing radiologists in area under the curve (AUC) and cutting UK reader workload by 88% [80]. The AI-STREAM trial in South Korea further confirmed generalisability: AI-assisted single reading improved cancer detection by 13.8% (5.70 vs 5.01 per 1000 screens) without increasing recall rates [23]. In Sweden’s paired-reader trial, substituting one radiologist with AI achieved comparable or superior detection (261 vs 250 cases; relative proportion 1.04; 95% CI: 1.00–1.09) [81]. Workflow simulations based on 249,402 mammograms projected 50% fewer double reads, +0.36% PPV, −0.88% arbitrations, and −0.59% benign biopsies, without compromising sensitivity or specificity [82].

### AI-stratified diagnosis: reducing low-value biopsies

AI supports more precise decisions around low-suspicion lesions—especially BI-RADS 4A, where benign outcomes are frequent. In the intelligent-augmented breast cancer risk calculator (iBRISK), biopsy PPV spanned 85.9% → 0.16% across AI risk deciles; applying these thresholds prospectively could avoid ~50% of unnecessary biopsies while maintaining cancer sensitivity [83]. A deep learning model trained on 288,767 ultrasound exams (> 5 million images) achieved AUC 0.976 in internal validation. In reader studies, it outperformed radiologists (AUC 0.962 vs 0.924) and reduced false positives by 37.3% and biopsy recommendations by 27.8%, without compromising sensitivity [84]. Convolutional neural network (CNN)‑based nomograms integrating clinical and ultrasound features have enabled the safe downgrading of BI‑RADS 4A lesions: in a cohort of 458 cases, 59.6% were reclassified as low risk, with malignancy rates falling to 1.5%, sensitivity maintained at 99.6%, specificity rising (4.4% → 45.3%), and overall accuracy improving (63.4% → 78.8%) [85]. Similarly, a multi-task deep learning model across four centres lifted the negative predictive value of BI RADS 4A lesions to 98.1% (*n* = 6420 exams), enabling surveillance rather than biopsy for most low-risk nodules [86]. Additional real-world evidence (e.g. PRAIM study; see “AI-augmented screening: improving accuracy and efficiency”) shows that AI-supported single-reader screening maintains PPV while reducing benign recalls/biopsies [85]. Retrospective simulations further support this: an AI-based triage system safely redirected 60–80% of mammograms to “no-read” streams, identifying up to 35% of future cancers within the high-risk group and potentially halving radiologist workload, while avoiding unnecessary reads and downstream biopsies in low-risk patients [24].

### AI-driven risk forecasting and personalised follow-up

Beyond immediate lesion assessment, AI increasingly supports longitudinal risk forecasting to personalise screening intervals and modalities. Multiple studies have demonstrated that AI-enhanced modelling surpasses traditional clinical tools in predicting future breast cancer risk. For example, Vachon et al integrated AI-derived risk scores with volumetric breast density to improve the prediction of advanced cancers 2–5.5 years after a negative mammogram (AUC 0.624 → 0.679; *p* = 0.01) [87]. Similarly, Arasu et al showed that five mammogram-based AI models outperformed the BCSC clinical model (AUC 0.63–0.67 vs 0.61), and that combining both approaches further raised AUCs to 0.66–0.68 [88]. A systematic review confirmed that image-only AI risk models consistently outperform traditional density- or history-based approaches (AUC 0.62–0.90 vs 0.54–0.69) [89]. In real-world cohorts, the deep learning model Mirai achieved 5-year AUCs of 0.65–0.71, with particular advantages for predicting low- and intermediate-grade malignancies [90]. Lauritzen et al further demonstrated that a hybrid AI + texture model (AUC 0.73) concentrated 44% of interval cancers within the top 10% of predicted risk [91], while Gjesvik et al validated commercial AI scores for risk stratification up to six years pre-diagnosis [92]. Retrospective modelling across >120,000 mammograms found the top 10% AI-risk group contained 86.8% of cancers, while the lowest-risk 70% accounted for just 4.4%, confirming AI’s potential for stratified follow-up [93].

### Implementation challenges and limitations

Despite encouraging results from Denmark, MASAI, PRAIM, and multinational studies, scaling AI in routine screening faces technical, human, and organisational barriers. Domain shift across vendors, protocols, and populations requires local calibration and prospective validation [94], while dataset imbalance necessitates subgroup reporting and fairness audits [95–97]. Performance gains often concentrate in high-confidence cases and may degrade near decision thresholds unless confidence-aware interfaces are used. Adoption is limited by practical constraints: PACS/electronic health record integration, legal liability, and governance of reading protocols [98, 99]. Programmes should continuously track recall, PPV, and interval cancer rates with periodic model recalibration [22, 100], and assess context-dependent economics, since baseline recall, workforce capacity, implementation costs, and savings from fewer false positives determine value [101, 102].

## Future perspectives

The rapid advancement of AI presents unprecedented opportunities to transform breast cancer screening through precision and efficiency. To strike a balance between early detection and minimising harm, several strategies are recommended:

1. Evidence-based recall targets: screening programmes should set recall rate benchmarks tailored to their population and adhere to evidence-based thresholds rather than imported norms. Regular audits can identify radiologists with recall rates outside target ranges and provide feedback.
2. Refinement of BI-RADS: research should focus on quantitative markers and AI augmented classification to refine BI-RADS 3 and 4A categories. Standardising follow-up intervals for probably benign lesions and adopting watchful-waiting strategies may reduce unnecessary biopsies.
3. AI Development and validation: AI algorithms should be trained and validated on diverse datasets, report transparency metrics and prospective trials and regulatory evaluation. Human–AI interaction should be studied to optimise trust and mitigate automation bias.
4. Patient-centred communication: improve patient-centred communication about screening. Educating women on the potential risks (over-detection and overtreatment), as well as the benefits of screening, can facilitate shared decision-making. Clear and culturally sensitive communication strategies may reduce anxiety and ensure management plans align with patient preferences.

## Conclusion

Over-detection and over-surveillance represent significant, multifaceted challenges in breast cancer screening, arising from oversensitive imaging, subjective classification systems, and a prevailing culture of caution. Recognising regional variation in recall rates and adopting evidence-based thresholds can help mitigate unnecessary detection, while attention must also be paid to the psychological, economic, and environmental consequences of excessive imaging. AI holds considerable promise: it enhances accuracy, improves risk stratification, and reduces clinical workload. Nevertheless, its implementation must be supported by robust validation, active bias mitigation, transparent performance reporting, and ethical oversight. Ultimately, a patient-centred, evidence-driven strategy that integrates technological innovation while carefully considering potential harms will deliver the greatest net benefit in breast cancer screening.

## Data Availability

Data sharing is not applicable to this article as no new datasets were generated or analysed during the current study.

## Abbreviations

AI: Artificial intelligence
AUC: Area under the curve
BCSC: Breast Cancer Surveillance Consortium
BI-RADS: Breast imaging reporting and data system
CI: Confidence interval
CO₂e: Carbon dioxide equivalent
DBT: Digital breast tomosynthesis
DM: Digital mammography
MASAI: Mammography screening with artificial intelligence (trial)
MRI: Magnetic resonance imaging
OR: Odds ratio
PERSPECTIVE I&I: PERSPECTIVE Integration & Implementation
PPV: Positive predictive value
PRAIM: Population-based real-world AI mammography (study)
